# Determining factors for the prevalence of anemia in women of reproductive age in Nepal: Evidence from recent national survey data

**DOI:** 10.1371/journal.pone.0218288

**Published:** 2019-06-12

**Authors:** Sujan Gautam, Haju Min, Heenyun Kim, Hyoung-Sun Jeong

**Affiliations:** Department of Health Administration, Graduate School, Yonsei University, Wonju City, Gangwon-do, Korea; Anglia Ruskin University, UNITED KINGDOM

## Abstract

Anemia is a condition in which the number of red blood cells is not sufficient to meet the physiological need of the body. Women of reproductive age and pregnant women are at a high risk of anemia, which in turn may contribute to maternal morbidity and mortality. We aimed to describe the prevalence of anemia and the factors associated with the risk of developing anemia in women of reproductive age in Nepal. Additionally, we examined the association of women’s decision-making autonomy regarding healthcare and experience of intimate partner violence (IPV) with anemia. Data from the 2016 Nepal Demographic and Health Survey (NDHS) were used in this study. The data were adjusted for sampling weight, stratification, and cluster sampling design. A battery-operated portable HemoCue was used to measure hemoglobin and detect anemia. Using complex sample logistic regression, the association between dependent and independent variables were examined; crude and adjusted odds ratio were reported. The mean (± SD) hemoglobin concentration was 12.13 g/dL (± 1.48). Overall, about 41% (95% CI 38.6–43.0%) of women aged 15–49 years were anemic. Women in households with wells as the source of drinking water (aOR 1.93; 95% CI 1.58–2.37) were significantly associated with an increased risk of developing anemia. While women who were currently using hormonal contraceptives (aOR 0.63, 95% CI 0.52–0.76) were significantly less likely to be anemic. After adjusting for background characteristics among women who were married at the time of the survey, decision-making autonomy regarding healthcare, and experience of IPV did not have a significant association with anemia. The high prevalence of anemia suggests the need for substantial improvement in the nutritional status of women. The increased disease burden compared with the past survey highlights the needs to reconsider the existing nutritional policy in Nepal.

## Introduction

Anemia or low concentration of hemoglobin (Hb) is a condition in which the number of red blood cells of the body is insufficient to meet physiological needs [1]. Iron deficiency is thought to be the most common cause of anemia worldwide. Children, women of reproductive age, and pregnant women are at high risk of developing anemia [2, 3]. Maternal anemia is associated with maternal and child morbidity and mortality such, as increased risk of miscarriage, stillbirth, prematurity, and low birth weight of the baby [4]. About 20% of perinatal mortality and 10% of maternal mortality in developing countries is attributed to iron deficiency [5].

In 2005, globally, 1.62 billion people were estimated to be affected by anemia [2]. Similarly, as per the 2011 estimates, the global prevalence of anemia among pregnant women is about 38% (32.4 million pregnant women), and non-pregnant women is about 29% (496.3 million non-pregnant women)), and for all women of reproductive age is about 29% (528.7 million women of reproductive age) [3]. The prevalence of anemia among non-pregnant and pregnant women is relatively low in high-income countries compared with low-and-middle-income countries [6]. The World Health Organization (WHO) classified the public health significance of anemia based on the prevalence estimated from blood levels of hemoglobin (normal, <4.9%; mild, 5.0–19.9%; moderate, 20.0–39.9%; and severe, ≥40.0%) [1].

Numerous factors including age, sex, residential elevation (altitude), smoking behavior, and pregnancy status influence hemoglobin concentration [1]. In addition to being a medical condition, anemia is an important socio-economic issue given its association with decreased physical and cognitive productivity [7]. A complex interplay of political, ecological, social, and biological factors determines the prevalence and distribution of anemia in a population [8]. Previous studies have documented several potential causes of anemia among women including rural residency [8], younger age [9], pregnancy status [10, 11], lower nutritional status [9], repeated childbearing [12], lactation/breastfeeding [9, 10], poor access to nutritional supplements during pregnancy [9], and exposure to domestic violence [13]. Additionally, helminths infection [14] and malaria [15] were found to be important causes of anemia. Furthermore, anemia was found to be associated with immunologic disease progression and increased risk of AIDS-related death [16]. On the contrary, the use of hormonal contraceptives was shown to have a potential protective effect against anemia [17].

Research on anemia in Nepal has mostly focused on adolescents [18–21], pregnant [22], and relatively younger women (aged 13–35 years) [23]. A majority of these studies are small-scale and are limited to a specific region. Moreover, the association of women’s decision-making autonomy and violence with the prevalence of anemia has not been investigated yet. The Nepal Demographic and Health Surveys (NDHS) is the only source of national data on several characteristics of women of reproductive age (15–49 years). Therefore, we designed this study to determine the prevalence of anemia and the factors associated with the risk of developing anemia among women of reproductive age in Nepal. Additionally, we examined the association of women’s decision-making autonomy regarding healthcare and experience of intimate partner violence (IPV) with anemia.

## Materials and methods

### Study design

In this study, we used the data from the 2016 NDHS, a nationally representative cross-sectional survey conducted as a part of Demographic and Health Surveys (DHS) by New ERA under the guidance of Ministry of Health, Nepal, which aims to provide up-to-date estimates of the basic demographic and health indicators [24]. The dataset of this survey was accessed publicly from ‘The DHS Program’ website upon subsequent registration and authorization [25].

Details of the questionnaires, procedures, and methodology used in the survey can be found elsewhere [24, 25]. Briefly, the 2016 NDHS was conducted based on the multi-stage cluster sampling technique. For sampling, each of the seven provinces of Nepal was divided into rural and urban area yielding 14 sample strata. The 2016 NDHS sample was then selected in two and three stages in rural and urban areas, respectively. In the first stage, 383 wards (urban, 184; rural, 199) were selected using the probability proportional to size sampling technique. In rural areas, wards were smaller and thus, served as the primary sampling unit (PSU); in urban areas, the wards were larger, and therefore, one enumeration areas (EAs) was randomly selected form each sampled ward in the second stage of sample selection. In the final stage, a fixed number of 30 households per cluster were selected using an equal probability systematic selection technique.

The 2016 NDHS used the following six questionnaires to collect data: household questionnaire, woman’s questionnaire, man’s questionnaire, biomarker questionnaire, fieldworker questionnaire, and the verbal autopsy questionnaire. The household questionnaire intended to provide information on the household characteristics of all members of the household. The women’s questionnaire was used to collect information from all women aged 15–49 years. The biomarker questionnaire was used to record height, weight and hemoglobin concentration and was provided only to the women in the subsample of the households selected for the men’s questionnaire. The data on anthropometric measures (height and weight) and hemoglobin concentration were first recorded on the paper during data collection and then entered into the computer-assisted system. The fieldworker questionnaire was used as a tool for examining data quality, and the verbal autopsy questionnaire was used for recording neonatal deaths.

### Measurement of variables

#### Dependent variable (anemia)

Blood samples for the hemoglobin test were collected from women who voluntarily provided their consent to undertake the test and otherwise excluded. Following a finger-prick, blood was drawn into a microcuvette for on-site analysis using a battery-operated portable HemoCue analyzer [24]. According to the WHO, for non-pregnant women aged ≥15 years, any anemia was defined as blood hemoglobin level <12.0 g/dL, which was further categorized as mild (11.0–11.9 g/dL), moderate (8.0–10.9 g/dL), and severe anemia (<8.0 g/dL); for pregnant women, any anemia was defined as blood hemoglobin level <11 g/dL, and further categorized as mild (10.0–10.9 g/dL), moderate (7.0–9.9 g/dL), and severe anemia (<7.0 g/dL) [1]. Hemoglobin concentration was adjusted for cigarette smoking and altitude (1000 m above sea level) [24]. For analysis, the severity of anemia was categorized as any-anemia and no-anemia.

#### Independent variables (predictors of anemia)

Predictors of anemia were chosen based on the array of literature pertaining to the risk of development of anemia among women in low-and-middle income countries including Nepal [8, 9, 11–13, 26–30].

The independent variables in this study included socio-demographic factors (age, ethnicity, place of residence, education level, occupation, household wealth status, and sources of drinking water); reproductive characteristics (fertility status, age at first birth, total number of children ever born, adverse pregnancy outcome for last/most recent pregnancy, birth in the past three years, breastfeeding status at the time of the survey, contraception use at the time of the survey, and iron supplementation during the most recent pregnancy in the past five years); nutritional and behavioral factors (body mass index (BMI) and cigarette/tobacco smoking); women’s autonomy in decision-making regarding her healthcare; and experience of any intimate partner violence (IPV; physical and/or sexual).

In this study, the self-reported age of the women, a continuous variable, was categorized into the following age groups: 15–24 years, 25–34 years, and 35–49 years [9]. Ethnicity was categorized as Brahmin/Chhetri, Janajati/Indigenous (includes Newar), Dalit and other castes (includes all other recorded ethnicities). Education level of women was categorized as no education (no schooling), primary (up to grade 5), secondary (up to grade 10), and higher (higher than secondary level). Household wealth status was determined by using the scores derived from the principal component analysis of several possessions, assets, and amenities the households own [31]. this derived variable was already included in the dataset as five quintiles ranked as poorest, poorer, middle, richer and richest each comprising 20% of the population [24]. In this study, wealth status was re-categorized as poor (includes poorest and poorer), middle, and rich (includes richer and richest) [32]. Occupation was categorized as did not work, professional/service (included technical, managerial, sales, and clerical), agriculture, and manual (included skilled and unskilled manual work) [33]. The sources of drinking water were grouped as tap water (public and private tap), well (tube well, dug well, borehole), surface water (river, dams, ponds, streams, springs) and others.

Fertility status of women was categorized as infecund/menopausal, fecund, pregnant, and postpartum amenorrhoeic. Women who were pregnant at the time of the survey were categorized as pregnant women. Women whose period has not returned since the last birth were categorized as postpartum amenorrhoeic women. Similarly, women who were not pregnant and not postpartum amenorrhoeic, and did not have a period in the last six months were categorized as menopausal women. Likewise, women were categorized as infecund, if they were not menopausal, not postpartum amenorrhoeic, not pregnant, and had no birth in the past five years. All other women who were not included in the former categories were categorized as fecund.

Adverse pregnancy outcome for the last/most recent pregnancy the women had was categorized as no (live birth) and yes (stillbirth, miscarriage, abortion). Contraception use was categorized as not using any method, hormonal (included pills, injections, implants, IUD, emergency contraception), female sterilization, male contraception (included male condoms and male sterilization), and traditional (withdrawal, abstinence, and other traditional methods). Iron supplementation during last pregnancy (for women with a live birth during the last five years of the survey) was categorized as not taken at all, took for <180 days, and took for ≥180 days. Women are recommended to take iron/folic acid supplements daily for a minimum of 180 days during pregnancy and until 45 days after childbirth [34].

Height and weight of all eligible women aged 15–49 years were measured by trained field staffs. BMI was calculated as the ratio of weight (in kilograms) to the square of height (in meters); it was recorded as a continuous variable and was available in the 2016 NDHS dataset [24]. For this study, BMI was categorized as underweight (less than 18.5 kg/m^2^), normal (18.5–24.9 kg/m^2^), overweight (25.0–29.9 kg/m^2^), and obese (≥30.0 kg/m^2^) [35]. For regression analysis, overweight and obese categories were merged into a single category as overweight/obese.

The 2016 NDHS collected information on domestic violence and women’s participation in household decision-making, specifically among currently married women. Women’s decision-making autonomy regarding her healthcare was categorized as high autonomy (if she takes a decision independently), medium autonomy (if she takes a decision with the partner), and low autonomy (if she is not involved). Women’s experience of intimate partner violence (IPV) was measured by analyzing two forms of violence, physical and sexual IPV. Physical IPV was determined based on women’s responses to seven questions by asking them whether their husbands ever did the following: 1. pushed, shook or thrown something at her; 2. slapped her; 3. twisted her arm or pulled her hair; 4. punched her with fist or something that could hurt her; 5. kicked, dragged or beat her; 6. tried to choke or burn her on purpose; and 7. threatened or attacked her with knife, gun, or any other weapon. Similarly, sexual IPV was determined based on women’s responses to three questions by asking them whether their husbands ever did the following: 1. physically forced her to have unwanted sexual relationships with him; 2. physically forced her to perform any other unwanted sexual acts; and 3. forced her with threats and any other way to performs unwanted sexual acts. A composite dichotomous summary was developed from 10 questions (physical IPV, 7 questions; sexual IPV: 3 questions) with yes/no answers to capture the women’s experience of any IPV and categorized as ever experienced IPV (‘yes’ response to at least one of the ten questions) and not experienced IPV (‘no’ response to all the ten questions). The Cronbach’s alpha for any IPV (10-item scale) was 0.876.

### Data analysis

The outcome variable of this study was the presence of anemia among women. a binary variable was created as any-anemia (mild, moderate, and severe) and no-anemia. Data analysis was carried out on SPSS 25.0 (IBM Corp., Armonk, NY). Due to the non-proportional allocation of the sample in the 2016 NDHS, the data were adjusted before any statistical analysis for sampling weights, stratification, and multistage sampling procedure to provide population-level estimates [36].

Data were analyzed using descriptive statistics to describe the characteristics of the study population. Frequencies and proportions (weighted) were reported. Continuous variables were reported using mean and standard deviation (SD). Chi-square test (χ^2^) or Fisher’s exact was used to assess the frequency distribution and the relationship between independent variables and anemia status. Similarly, bivariate logistic regression analysis was used to determine the individual effect of each factor on anemia status. Finally, multivariate regression analysis model was performed to determine the adjusted effect of each factor on the dependent variable. The results of regression analysis were presented by crude/unadjusted odds ratio (OR) and adjusted odds ratio (aOR) with 95% confidence intervals (CIs). A variable with a p-value <0.05 in the bivariate analysis were considered statistically significant and thus, included in the final regression model. Prior to the multivariate regression analysis, the independent variables were checked for multicollinearity by examining the variance inflation factor (VIF); no serious issues (VIF≤ 2) were found [37].

Additionally, a separate multivariate regression analysis model was generated to find out the impact of women’s decision-making and experience of IPV on anemia status; a similar data analysis procedure was followed.

### Ethical approval

As the study was based on secondary data from the 2016 NDHS, no separate ethical approval was sought. Nevertheless, the 2016 NDHS was reviewed and approved by the ICF International Review Board and the Nepal Health Research Council (NHRC). Details on ethical procedures used in the DHS survey can be found elsewhere [38].

## Results

### Baseline demographic characteristics of participants

The baseline demographic characteristics of the study population are shown in Table 1. A total of 6,414 women aged 15–49 years (mean [±SD] age, 29.2 years [±9.6]) were included in the study. The highest proportion of the women were from the 15–24 years age group (38%), from janajati ethnicity (36%), residing in urban area (63%), had secondary education (35%), was involved in agriculture (47%), belonged to rich family (43%), and used tap water (48%). The majority of the women were fecund (79%), had not given birth in the past three years (78%), were not breastfeeding (78%), and were not using any contraceptive (60%). About one-third of the women (33%) had ≥3 children. Of the 4,572 women with children, the mean (± SD) age at first birth was 19.7 years (± 3.2), and the majority of them (54%) were <20 years when they gave the first birth. Of the 4,651 women who were ever pregnant, the majority (87%) had no adverse pregnancy outcomes in their last/most recent pregnancy. Of the 2,014 women who had a live birth in the past five years, about two of the five women (40%) took iron supplements for >180 days during their last pregnancy. The mean (±SD) BMI was 22.2 (±4.0) and approximately three of the five women (61%) had a normal BMI. The majority of the women (91%) were non-smokers.

**Table 1:** Prevalence of anemia based on the baseline demographic characteristics of women of reproductive age in Nepal.

Baseline demographic characteristics	Total (Weighted)^#^	n (%) ^#^	Any Anemia^b^ n (%) ^#^ [Non-pregnant: Hb<12 g/dL, Pregnant: Hb<11 g/dL]	p-value
**Age**	6414	(29.23 ± 9.69)^a^		<0.001
15–24		2443 (38.1)	1065 (43.6)	
25–34		1971 (30.7)	814 (41.3)	
35–49		2000 (31.2)	735 (36.8)	
**Ethnicity**	6414			<0.001
Brahmin/Chhetri		2015 (31.4)	735 (36.5)	
Janajati/Indigenous		2335 (36.4)	885 (37.9)	
Dalit		803 (12.5)	308 (38.4)	
Other castes		1262 (19.7)	686 (54.4)	
**Place of residence**	6414			0.20
Rural		2385 (37.2)	1018 (42.7)	
Urban		4029 (62.8)	1596 (39.6)	
**Education**^**c**^	6414			0.08
No education		2144 (33.4)	892 (41.6)	
Primary		1069 (16.7)	411 (38.4)	
Secondary		2277 (35.5)	972 (42.8)	
Higher		924 (14.4)	339 (36.7)	
**Occupation**	6414			0.18
Did not work		2096 (32.7)	899 (42.9)	
Professional/Service/Sales		955 (14.9)	359 (37.6)	
Agriculture		2994 (46.7)	1218 (40.7)	
Manual (skilled/unskilled)		370 (5.8)	138 (37.4)	
**Household Wealth status**^**d**^	6414			<0.001
Poor		2318 (36.1)	862 (37.2)	
Middle		1317 (20.5)	645 (49.0)	
Rich		2779 (43.3)	1107 (39.8)	
**Sources of drinking water**	6414			<0.001
Tap		3078 (48.0)	989 (32.1)	
Well		2534 (39.5)	1340 (52.9)	
Surface (river/spring)		280 (4.4)	88 (31.4)	
Others		522 (8.1)	197 (37.7)	
**Fertility status**	6414			
Infecund, menopausal		747 (11.7)	278 (37.2)	0.018
Fecund		5074 (79.1)	2057 (40.5)	
Pregnant		290 (4.5)	133 (46.0)	
Postpartum amenorrhoeic		303 (4.7)	145 (47.9)	
**Age at first birth**	4572	(19.72 ± 3.26)^a^		P = 0.14
<20 years		2467 (54.0)	1033 (41.9)	
20–25 years		1732 (37.9)	679 (39.2)	
≥25 years		373 (8.2)	132 (35.4)	
**Total number of children ever born**	6414			0.35
No children		1842 (28.7)	770 (41.8)	
1–2 children		2432 (37.9)	957 (39.4)	
≥3 children		2140 (33.4)	887 (41.4)	
**Adverse Pregnancy outcome for last/most recent pregnancy**	4651			0.19
No (live births)		4042 (86.9)	1650 (40.8)	
Yes (stillbirths, miscarriage, abortion)		609 (13.1)	230 (37.8)	
**Birth in the past 3 year**	6414			<0.001
No		5053 (78.5)	1975 (39.2)	
Yes		1379 (21.5)	640 (46.4)	
**Currently breastfeeding**	6414			<0.001
No		4988 (77.8)	1961 (39.3)	
Yes		1426 (22.2)	653 (45.8)	
**Current contraception use**	6414			<0.001
Not using		3832 (59.7)	1625 (42.4)	
Hormonal		9.6 (14.1)	267 (29.5)	
Female sterilization		730 (11.4)	392 (53.7)	
Male contraception		460 (7.2)	142 (30.9)	
Traditional		486 (7.6)	189 (38.9)	
**Iron/folic acid supplementation during last pregnancy**	2014			0.347
Not at all		195 (9.7)	93 (47.7)	
<180 days		1012 (50.2)	455 (45.0)	
≥180 days		807 (40.1)	339 (42.0)	
**BMI**^**e**^	6411	(22.21 ± 4.04)^a^		<0.001
Underweight		1077 (16.8)	518 (48.1)	
Normal		3925 (61.2)	1673 (42.6)	
Overweight		1087 (17.0)	333 (30.6)	
Obese		322 (5.0)	90 (28.0)	
**Cigarette/Tobacco smoking**	6414			<0.001
Smoker		573 (8.9)	166 (29.0)	
Non-smoker		5841 (91.1)	2449 (41.9)	
**Hemoglobin Level**^**f**^ **(g/dl)**	6414	(12.13 ± 1.48)^a^		
**Anemia Status**	6414			
No anemia		3800 (59.2%)		
Mild anemia		2147 (33.5%)		
Moderate anemia		450 (7.0%)		
Severe anemia		17 (0.3%)		
*Any Anemia*^**g**^		*2614 (40*.*8)*		

n: number. %: percentage. Hb: hemoglobin. BMI: body mass index.

^#^The numbers and percentages are adjusted for multi-stage sampling, cluster weight, and sample weight.

^a^(Mean ± SD).

^b^(mild, moderate, and severe anemia combined).

^c^Education level was classified as no education (no years of schooling), primary (up to grade 5), secondary (up to grade 10), and higher (higher than secondary level).

^d^Household wealth status was determined using scores derived from principal component analysis of various household possessions, assets, and amenities.

^e^BMI was categorized as underweight (<18.5 kg/m^2^), normal (18.5–24.9 kg/m^2^), overweight (25.0–29.9 kg/m^2^), and obese (≥30.0 kg/m^2^).

^f^Hb level adjusted for altitude and smoking.

^g^Includes the sum of mild, moderate and severe anemia.

### Prevalence of anemia

The mean (±SD) hemoglobin level concentration was 12.1 g/dl (±1.4). An overall prevalence of any anemia among women aged 15–49 years was 41% (95% CI, 38.6%–43.0%). Specifically, mild, moderate, and severe anemia was found in 33% (95% CI 31.5%–35.5%), 7% (95% CI 6.2%–7.9%), and 0.3% (95% CI 0.1%–0.5%) of the women, respectively. The prevalence of anemia decreased with the increase in both the age group (i.e., 44% among women aged 15–24 years vs. 37% among women aged 35–49 years), and age at first birth (42% among women who had first birth <20 years compared to 35% among women who had first birth ≥25 years). The highest prevalence of anemia was found among women: residing in rural area (43%), having secondary education (43%), unemployed (43%), of middle-class family (49%), having wells as the sources of drinking water (53%), postpartum amenorrhoeic (48%), had birth in the past three years (46%), breastfeeding (46%), not using any contraceptive methods (42%), underweight (48%), and non-smoker (42%) (Table 1).

### Factors associated with the risk of developing anemia among women of reproductive age

Table 2 shows the unadjusted and adjusted logistic regression analyses of the factors associated with the risk of developing anemia among women of reproductive age. Having well water as the source of drinking water (aOR 1.93; 95% CI 1.58–2.37) was significantly associated with an increased risk of developing anemia. Compared to women who did not use any contraceptives, women who used hormonal contraceptives (including intrauterine devices) had reduced odds of developing anemia by 37% (aOR 0.63; 95% CI 0.52–0.76) while, having permanent sterilization was associated with a 146% increase in odds of developing anemia (aOR 1.46; 95% CI 1.20–1.78). Overweight/obese women had reduced odds of developing anemia (aOR 0.64; 95% CI 0.51–0.79) compared to women with normal body weight. Similarly, women who were smoker had lower odds of developing anemia (aOR 0.68; 95% CI 0.55–0.84) compared to non-smoker women. Factors such as age, ethnicity, household wealth status, fertility status, having a birth in the past three years, and breastfeeding did not reveal any significant association with the risk of developing anemia despite their significant relationship in the univariate analysis.

**Table 2:** Factors associated with the risk of developing anemia among women of reproductive age in Nepal (n = 6410)^a^.

	Unadjusted/Crude		Adjusted^b^	
Variables	OR (95% CI)	p-value	OR (95% CI)	p-value
**Socio-demographic information**				
**Age (years)**		<0.001		0.403
35–49	Reference		Reference	
25–34	1.21 (1.04–1.39)		1.07 (0.90–1.27)	
15–24	1.32 (1.15–1.52)		1.15 (0.95–1.30)	
**Ethnicity**		<0.001		0.658
Brahmin/Chhetri	Reference		Reference	
Janajati/Indigenous)	1.06 (0.88–1.27)		1.01 (0.85–1.20)	
Dalit	1.08 (0.82–1.43)		0.90 (0.71–1.15)	
Other castes	2.09 (1.67–2.61)		1.09 (0.84–1.42)	
**Household wealth status**		<0.001		0.356
Poor	Reference		Reference	
Middle	1.63 (1.38–1.91)		1.12 (0.94–1.33)	
Rich	1.11 (0.94–1.32)		0.98 (0.81–1.19)	
**Sources of drinking water**		<0.001		<0.001
Tap	Reference		Reference	
Well	2.36 (1.98–2.80)		1.93 (1.58–2.37)	
Surface (river/spring)	0.97 (0.71–1.33)		0.93 (0.68–1.27)	
Others	1.26 (0.96–1.63)		1.17 (0.90–1.52)	
**Reproductive information**				
**Fertility Status**		0.032		0.639
Infecund, menopausal	Reference		Reference	
Fecund	1.14 (0.93–1.39)		1.13 (0.92–1.39)	
Pregnant	1.43 (1.04–1.97)		1.21 (0.87–1.70)	
Postpartum amenorrhoeic	1.56 (1.12–2.16)		1.15 (0.79–1.65)	
**Birth in the past 3 year**		<0.001		0.128
No	Reference		Reference	
Yes	1.34 (1.16–1.55)		1.25 (0.93–1.66)	
**Currently breastfeeding**		<0.001		0.762
No	Reference		Reference	
Yes	1.30 (1.13–1.50)		1.04 (0.78–1.38)	
**Current contraception use**		<0.001		<0.001
Not using	Reference		Reference	
Hormonal	0.58 (0.49–0.69)		0.63 (0.52–0.76)	
Female sterilization	1.59 (1.34–1.89)		1.46 (1.20–1.78)	
Male contraception	0.61 (0.45–0.84)		0.80 (0.57–1.16)	
Traditional	0.86 (0.69–1.07)		0.97 (0.77–1.22)	
**Nutritional and behavioral characteristics**				
**BMI**		<0.001		<0.001
Normal	Reference		Reference	
Underweight	1.25 (1.07–1.46)		1.07 (0.91–1.26)	
Overweight/obese	0.59 (0.48–0.72)		0.64 (0.51–0.79)	
**Cigarette/Tobacco smoking**		<0.001		<0.001
Non-smoker	Reference		Reference	
Smoker	0.58 (0.47–0.71)		0.68 (0.55–0.84)	

^a^Adjusted model consisted of 6,410 observations due to some missing values.

^b^Only variables with p<0.05 are retained in the adjusted multivariate model.

OR: odds ratio. CI: confidence interval. All values are weighted for the multi-stage sampling, cluster weight, and sampling weight.

### Decision-making autonomy, IPV, and anemia in married women

Of the 3667 women who were married or in-union women at the time of the survey, more than one-third (37.8%) were not involved in decision-making regarding their healthcare and less than one-fourth (23.9%) have ever experienced physical or sexual IPV. In the unadjusted model, women who took the decision jointly with their partners regarding her healthcare and who experienced IPV were significantly associated with the increased risk of anemia. However, in the multivariate analysis, after adjusting the model for background characteristics (socio-demographic, reproductive, nutritional and behavioral characteristics), women’s decision-making autonomy regarding her healthcare and experience of IPV were not significantly associated with an increased risk of developing anemia (Table 3).

**Table 3:** Association of decision-making autonomy and IPV with the risk of developing anemia among women of reproductive age (married/in-union) in Nepal (n = 3667)^a^.

Variables	Total n (%)	Any anemia n (%)	Unadjusted OR (95% CI)	p-value	Adjusted OR (95% CI)^b^	p-value
**Decision-making autonomy for own health care**				0.049		0.125
High autonomy	940 (25.6)	345 (36.7)	Reference		Reference	
Medium autonomy	1341 (36.6)	574 (42.8)	1.29 (1.04–1.59)		1.11 (0.89–1.39)	
Low autonomy	1386 (37.8)	545 (39.3)	1.19 (0.90–1.37)		0.92 (0.73–1.16)	
**Ever experienced any IPV**				0.001		0.180
Not-experienced	2790 (76.1)	1060 (38.0)	Reference		Reference	
Experienced	877 (23.9)	403 (46.0)	1.38 (1.15–1.67)		1.15 (0.93–1.42)	

n: number, %: percentage.

^a^Adjusted model consisted of married/in-union women (3667).

^b^Final model adjusted for age, ethnicity, household wealth status, drinking water source, maternity status, birth in the past three years, breastfeeding, contraception use, BMI, and cigarette/tobacco smoking.

OR: odds ratio. CI: confidence interval. All values are weighted for the multi-stage sampling, cluster weight, and sampling weight.

## Discussion

The 65^th^ World Health Assembly, 2012 approved an action plan and global targets for maternal and child health nutrition including infants, with a commitment to decrease the prevalence of anemia among women of reproductive age by to halve by 2025, from 2011 levels [6]. This study was carried out to understand the influence of a wide range of socio-demographic, reproductive, nutritional, and behavioral factors on the prevalence of anemia among women of reproductive age in Nepal. Additionally, we studied the role of women’s decision-making autonomy regarding her healthcare and experience of IPV on the risk of developing anemia. After controlling for available independent variables, women in a household with a well as the source of drinking water, and those who had undergone sterilization were more likely to be anemic. On the other hand, women who were using a hormonal contraceptive method, were overweight/obese and were smoking cigarette/tobacco were less likely to be anemic. These findings provide a national estimate of anemia among women of reproductive age in Nepal and could serve as a benchmark to evaluate the national nutrition programs.

This study found that two of the five women of reproductive age were anemic. Given the high prevalence (over 40%) identified in this study, anemia must be considered as a severe public health problem [1]. The prevalence of anemia revealed by this study is higher than both the national average (35.1%), and the world average (32.8%) [39]. On the other hand, some cross-sectional studies showed that the relatively low prevalence of anemia among younger women (aged 13–35 years) in Nepal could be due to the selection of apparently healthy women living in an urban setting and non-endemic areas for malaria and hookworm infestation [23]. Previous studies using DHS data on women of reproductive age, showed relatively lower prevalence of anemia in Nepal (34.4%) and higher prevalence in Pakistan (51.1%) [9] and Bangladesh (41.3%) [30], thereby, demonstrating a wide variation in the prevalence of anemia within similar regional settings of South-Asian countries. However, the difference in the prevalence may be attributed specifically to the different geographical, cultural, and dietary factors in these countries. High prevalence of anemia among women of developing countries reflect their social and biological vulnerability both within the household and society [40]. In developing countries, the major drivers of nutritional deficiency including nutrition-related anemia include poverty as well as one’s social position within the household [18]. Especially in South-Asia, gender inequality and lack of control by women over their lives underlie the lack of access to iron-rich food [41]. Nevertheless, recently, awareness of anemia and its consequences on the health of women and children has increased. Globally, the prevalence of anemia fell by 12% between 1995 and 2011 [4], indicating the possibility of its prevention and management. In the South-Asian region, a decreasing trend in the prevalence of anemia among women of reproductive age was observed between 1995 and 2011; however, the prevalence among pregnant women was almost unchanged [6]

There was no significant association of women’s age with being anemic. In contrary to our findings, other studies performed in developing countries of Africa and Asia, documented the association of women’s age and anemia [9, 11, 26, 27]. Prevalence of anemia was found to decrease with the increase in age. A higher prevalence of anemia among relatively younger women could be due to the adverse effect of lower dietary iron intake and the additional demand for iron imposed by iron loss during menstruation, pregnancy, and lactation [2]. This study also found that more than fifty percent of women were <20 years when they gave the first birth and the rate of anemia among them was also higher. Therefore, another possible reason for the high prevalence of anemia among younger women could be due to teenage pregnancy. Similarly, the likelihood of anemia was found to be increased with multiple pregnancies [12]. The physical burden of multiple pregnancies coupled with low-quality diets, parasitic infections, and poor sanitation poses a risk for the mother and the infant [9]. Likewise, the higher prevalence of anemia among lactating women can be attributed to the increased nutritional demand imposed by lactation [42].

The prevalence of anemia among poor and rural women was found to be relatively high. Though poverty affected women’s nutritional status [40], this study found no significant differences between anemia and household wealth status, consistent with another study from Bangladesh [30]. This study also found no significant association between ethnicity and anemia suggesting that ethnicity may not a determining factor for the prevalence of anemia. Women using wells as the source of drinking water were about two times more likely to be anemic as compared to those who use tap water. Terai region of Nepal is home to 48% of the total population. About 90% of the population living in this region use groundwater for domestic purpose including drinking [43]. The arsenic concentration is higher in groundwater, especially in shallow tube wells [44]. There is evidence of increased risk of anemia among women who had exposure to arsenic from drinking water [45]. In the past, Nepal had a relatively high rate of open defecation [46]. However, after the introduction of the government’s initiatives at reducing open defecation in 2005, a rapid improvement in the toilet coverage was observed [47]. Nevertheless, we cannot neglect the possibility of contamination of water in the wells with parasite, as most of the wells are uncovered and unprotected in Nepal. Basic hygiene has been documented to reduce the risk of parasitic infections and is considered as one of the interventions for prevention and control of anemia. Therefore, water and sanitation interventions play a vital role in reducing the nutritional losses caused by infection [4]. Combining iron supplementation with malaria prevention and treatment can be beneficial for improving the hemoglobin concentration [6]. Preventive interventions such as the use of insecticide-treated bed nets and intermittent preventive treatment in malaria-endemic regions have been found to improve hemoglobin concentrations of pregnant women [8]. Evidence also suggests that iron-folic acid supplementation and de-worming program have successfully reduced iron deficiency anemia including soil-transmitted helminth infection [48].

In this study, the prevalence of anemia was high among women who were pregnant (46%) and postpartum amenorrhoeic (48%). Previous studies have documented the significant association between pregnancy and anemia [10, 11], however, we did not find such association. The possible reason may be the categorization of the variables we used in our study. Unlike those studies, we did not categorize the pregnancy status of women as pregnant and no-pregnant; instead we considered the broad categories of the fertility status of women and categorized into four categories: infecund/menopausal, fecund, pregnant, and postpartum amenorrhoeic. Nevertheless, various food-based programs, including intermittent iron supplementation alone or together with other micronutrients can be used for high-risk groups especially women in childbearing age and pregnancy to improve dietary deficiency and as a preventive measure where parasitic infestations are prevalent [6]. The WHO recommends a daily dose of 30–60 mg of iron and 0.4 mg of folic acid supplementation as early as possible during pregnancy to reduce the risk of low birth weight, iron deficiency, and maternal anemia [49].

This study found a mixed relationship between anemia and type of contraceptive methods used by women. The highest prevalence of anemia was found among women who had permanent sterilization while the lowest prevalence was found among hormonal contraceptive users. Previous literature from Nepal found women using Depo-Provera injections had a significantly higher concentration of hemoglobin [23]. Consistent with these findings, a significant negative association was found between anemia and hormonal contraceptive use [17, 29], suggesting the potential protective effect of hormonal contraceptives against anemia. Reduction in menstrual bleeding associated with the use of hormonal contraceptives could be the plausible underlying mechanism [50]. Evidence of non-contraceptive benefits of hormonal and IUD contraceptive methods against menstrual blood flow and other women’s problems such as endometriosis, endometrial cancer, ovarian cancer, colorectal cancer, ectopic pregnancy, and pre-menstrual dysphoric disorder has been documented in the literature [51].

This study showed a high prevalence of anemia among underweight women. Similarly, overweight women were less likely to be anemic as compared with women with normal BMI. A study done in China revealed that overweight/obese women had higher iron consumption compared to women with lower BMI [52]. Having higher BMI was associated with less risk of developing anemia compared to those with lower BMI [53]. Similarly, the studies done in developing countries in South-Asia found that undernourished women were more likely to be anemic than women with higher BMI [9, 12, 30, 40, 54], suggesting a serious policy attention to include micronutrients initiatives as a prioritized program in this particular group. Women suffering from undernourishment are more likely to be deficient in essential micronutrients which may be associated with increased risk of anemia. In contrast, a meta-analysis showed obesity as an emerging risk factor for anemia [55].

The prevalence of anemia was high among women who were non-smokers compared with smokers, and the association was statistically significant. The gradual upward shift in the mean hemoglobin levels associated with increased number of cigarettes smoked per day seemed to reduce the utility of hemoglobin level to potentially detect anemia [56]. This might have contributed to the comparatively lower prevalence of anemia among smokers. In contrast, a study conducted in India showed higher rates of anemia among tobacco users and it was strongly positively associated with anemia [57].

Apart from investigating the prevalence of anemia among women of reproductive age, this study explored the role of women’s decision-making autonomy regarding her healthcare and experience of IPV with anemia. The association of women’s decision-making and IPV was somewhat attenuated after the adjustment with several characteristics.

The prevalence of anemia was found to be lower among married women who independently took the decision regarding her healthcare. This can be explained by the women’s relative position in the household in terms of decision-making. A woman’s autonomous decision-making is associated with increased utilization of maternal health services [58], thereby resulting in better health outcome. Women’s autonomy can build up women’s capacity to control resources and promote self-perceptions, self-confidence, a realization of rights and the ability to achieve them [59].

The prevalence of anemia was high in the women who experienced multiple instances of domestic violence. Women’s experience of IPV was found to be significantly associated with anemia in the unadjusted model but lost its significance when adjusted with other variables. Consistent with this finding, the study done in India showed a similar result of the association between the experience of domestic violence and anemia [13]. Literature has documented the association between women’s experience of domestic violence and increased risk of malnutrition [60]. Women exposed to economically abusive intimate interpersonal violence appeared to have food insecurity [61]. A study conducted in Nepal showed that women living in food-insecure households are more likely to experience some forms of IPV [62]. Similarly, domestic violence was strongly linked to women’s inability to take a decision regarding the type and quantity of food [60]. The withholding of food, as a form of abuse against women, results in the dietary inadequacy, which subsequently causes nutritional deficiencies including anemia [13]. Additionally, domestic violence, being a sensitive issue, is often underreported, which in turn, could affect the magnitude of its association with nutritional status of women. Women were less likely to report violence against them because of fear of husband or some family members [63], fear of losing family prestige as well as economic support from the family [64].

This study is based on the data from a large national survey with a high response rate. The data were adjusted for multistage sampling design used in the survey to make the findings of this study nationally reproducible. Nonetheless, there are some limitations of this study, therefore the findings should be handled with cautions. First, this study did not differentiate between different types of anemia because many other factors such as vitamin B-12, folate, ferritin, and parasitic infections were not included in this study. Additionally, this study did not consider the assessment of dietary factors that might potentially have an impact on the development of anemia. Finally, due to the cross-sectional design of the study, it was not possible to determine the cause-effect relationship between the predictor variables and anemia; further prospective research are required to understand this relationship.

## Conclusion

This study demonstrates the factors associated with the prevalence of anemia in Nepal. It is quite evident from the study findings that the general health status of the women of reproductive age in Nepal needs substantial improvement. This study found that about 41% of the women of reproductive age was anemic. Thus, anemia could be considered as a severe public health problem. The higher prevalence of anemia revealed in this study compared to the past survey highlights the needs to reconsider the existing nutritional policy in Nepal. The prevalence of anemia was higher among underweight women, while obese women were less likely to become anemic. Both, anemia and lower BMI are the indicators of poor nutritional status. Poor diet quality may result in the lower bioavailability of dietary iron, thus increasing the risk of developing anemia. An overall higher prevalence of anemia suggests the necessity of developing appropriate nutrition interventions; iron supplementation could be the immediate measure to cure anemia while periodic deworming, food diversification, and food fortification might be the long-term measures for the management of anemia. Given that a larger proportion of households are using well-water as the source of drinking water, appropriate measures to protect the well-water such as protecting the wells and disinfection of water before use may decrease the likelihood of parasite infestation, and in turn, reduce the prevalence of anemia. Mobilization of community health workers and volunteers can be a strategic measure to reach a large section of adolescents including women of reproductive age. Anemia surveillance and interventions programs specific to targeted groups and high-risk populations are recommended for reduction of anemia. Further research studies are advised to understand the prevalence and determinants of anemia.
